# Use of a community-led prevention strategy to enhance behavioral changes towards Ebola virus disease prevention: a qualitative case study in Western Côte d’Ivoire

**DOI:** 10.1186/s41256-017-0055-6

**Published:** 2017-12-22

**Authors:** Lara Gautier, Koffi Ange Houngbedji, Jeanne Uwamaliya, Megan Coffee

**Affiliations:** 1 Department of Social and Preventive Medicine, School of Public Health (ESPUM) University of Montreal, Québec, Canada; 2 Public Health Research Institute (IRSPUM), University of Montreal, Québec, Canada; 3 Centre d’Etudes en Sciences Sociales sur les Mondes Africains, Américains et Asiatiques, Université Denis Diderot, Paris-Sorbonne Cité, France; 4 International Rescue Committee, Abidjan, Côte d’Ivoire; 5 International Rescue Committee, 122 East 42nd Street, New York, NY 10168-1289 USA

**Keywords:** Community-led prevention; behavior change, Ebola virus, Côte d’Ivoire

## Abstract

**Background:**

Starting in December 2013, the Ebola virus disease (EVD) epidemic spread in West Africa through five countries (Sierra Leone, Liberia, Guinea, Nigeria, and Mali), killing over 11,300 people. In partnership with Côte d’Ivoire’s Ministry of Health, the International Rescue Committee instigated a community-led strategy aimed at promoting behavior change in order to prevent potential Ebola outbreaks in the country. The strategy was implemented in Western districts bordering Liberia, Guinea, and Mali. This study aims to analyze the community-led strategy, to document lessons learned from the experience, and to capitalize on the achievements.

**Methods:**

A case study in four districts of Western Côte d’Ivoire, i.e. Biankouma, Danané, Odienné and Touba districts was carried out. Qualitative data in 12 villages (i.e., three villages per district) was collected from 62 healthcare workers, community leaders, and ordinary community members. Data was de-identified, coded and analyzed using a thematic approach.

**Results:**

The community-led strategy was socially accepted in the villages. Even though some community leaders reported that sensitization had been, at times, constrained by a lack of equipment, the people interviewed demonstrated accurate understanding of information about prevention practices. Some practices were easily adopted, while others remained difficult to implement (e.g., ensuring safe and dignified dead body management).

**Conclusion:**

This research demonstrates that sensitization efforts led by well-integrated and respected community leaders can be conducive of behavior change. Lessons learned from the community-led strategy could be applied to future disease outbreaks.

## Background

Beginning in December 2013, the Ebola Virus Disease (EVD) epidemic in Western Africa grew rapidly, affecting primarily Guinea, Sierra Leone, and Liberia and killing over 11,300 people [1]. Due to its geographical proximity to affected countries, Côte d’Ivoire and particularly its Western regions were exposed. Responding to this new threat, the government of Côte d’Ivoire established a National Plan on EVD Prevention and Response in September 2014.

Coincidentally, the International Rescue Committee (IRC) brought technical support to strengthen the country’s capacities in terms of EVD prevention and control in four health districts of Western Côte d’Ivoire sharing a border with a country affected by EVD. The program included community sensitization, trainings on EVD prevention and rapid-intervention, implementing a community surveillance system using mobile phones, and building Ebola Treatment Centers and isolation units.

Communities are pivotal players in Ebola response plans [2–4]. In Côte d’Ivoire, by early 2015, over 1500 Community Health Workers (CHW) were responsible for collecting and reporting data on any suspected cases and events through mobile phones. Communities are also drivers of behavior change: IRC worked to empower local communities to identify unsafe practices and find culturally and socially acceptable replacement strategies. This initiative was called, the “community-led infection prevention” approach. It involved the creation of “monitoring committees” comprising CHWs, community leaders, representatives of youth, women, religious leaders, and traditional healers. Four activities were developed. First, EVD prevention messages had to be revised. Ebola-related official recommendations (enforced by governments’ authorities in order to reduce the burden of practices considered unsafe because they contributed to spread EVD [5]) were converted into recommendations promoting more socially and culturally acceptable practices (hence coined “alternative practices”) [6]. Table 1 outlines the correspondence between the two sets of practices

**Table 1:** Unsafe practices and proposed alternative practices

EXAMPLES OF UNSAFE PRACTICES FORBIDDEN BY GOVERNMENT’S AUTHORITIES	EXAMPLES OF ALTERNATIVE PRACTICES
Consumption of bushmeat	Consumption of fish and livestock or poultry (chicken, beef, mutton, goatmeat...)
Handshaking and hugging as a greeting	Raising arms as a greeting
Infrequent hand washing	Regular handwashing with water and soap or ash
Unsafe dead body management, including direct contact with biological liquids by multiple people close to the deceased	Inform the head of the health area, use chlorinated water during funeral baths Wear household gloves when touching the dead body Reduce the number of people in contact with the body
Use of the clothing of the deceased, including that worn during illness	Abstaining from wearing the clothing of the deceased
Having contact with newcomers coming from an Ebola-affected country	Inform the head of the health area of any strangers coming from an Ebola-affected country

Second, the initiative developed and disseminated locally-adapted communication tools (e.g., flipchart booklets). Third, it trained “monitoring committees” to spread sensitization messages. Fourth, it organized sensitization sessions near health centers, and public areas (e.g., markets). In total, 1930 members of “monitoring committees” were trained to spread culturally and socially appropriate messages to prevent EVD infection.

**Fig. 1:**
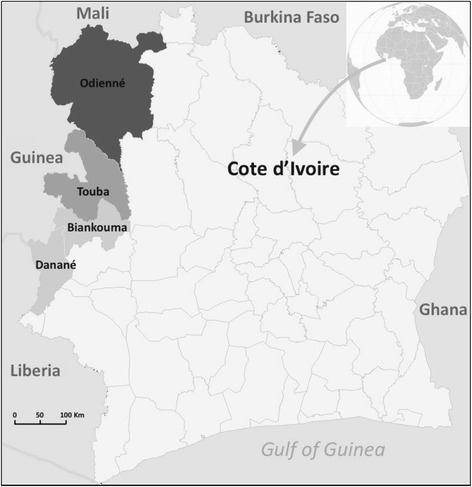
Map of the four districts of Côte d’Ivoire included in the study. NB: The districts of Biankouma and Danané belong to the same region, i.e. Tonkpi region. Source: Adapted from Map of the departments of Côte d’Ivoire (Ivory Coast). Created by Rarelibra in 2007 for public domain use, using MapInfo Professional v8.5 and various mapping resources. Available from: https://commons.wikimedia.org/wiki/File:Côte_d%27Ivoire_departments.png

The study was conducted in the four target districts: Biankouma and Danané, both located in Tonkpi region; and the districts of Odienné and Touba, located in Kabadougou and Bafing regions, respectively (see Fig. 1).

The experience of Côte d’Ivoire is remarkable: despite the proximity of the threat and the size of the epidemic, Ebola was not identified in Côte d’Ivoire during the West African outbreak. Reasons why Ebola did not spread in this country typically include: i) the limited number of cases in the rural areas of Guinea and Liberia across the border, ii) the administrative closure of the borders by Côte d’Ivoire authorities, iii) the fact that any cases that did enter did not spread the disease. However, it is interesting to look into how communities were prepared in an area that did not have Ebola spread, despite the long Côte d’Ivoire-Guinea-Liberia border.

Using a participatory approach, this study aims to draw lessons from implementation of the program, emphasizing the following aspects: i) people’s knowledge of unsafe practices, alternative practices, and effective behavior changes; ii) the role played by monitoring committee members, including evidence of leadership in identifying alternative practices and means of behavior change; iii) the effectiveness of using various sensitization methods; iv) the relevance of joint community-led strategies in allowing for the successful implementation of community surveillance system; and (v) capitalizing on achievements in the long term. Findings can inform future programs implemented to prevent viruses from spreading in Subsaharan Africa.

## Methods

A multiple case-study design [7] using a participatory approach was used.

### Sampling methods for selection of districts, villages, and people

The cases are the geographic entities that represent the four selected districts: Biankouma, Danané, Odienné and Touba. Data was collected in three villages in each of the four target districts. In total, 12 villages were selected.

In each selected village, data was collected through individual in-depth interviews with members of monitoring committees, ordinary community members, and CHW supervisors (i.e., qualified health workers). Data was also collected through focus group discussions with ordinary community members (4–8 participants, all members of the same community).

The selection of participants matched the aim of the research project – to collect information and experiences from the various actors involved in behavior change as a result of the Ebola epidemic. Diversification of data sources was sought: data was extracted from i) different geographical areas (according to their distance from the border), and ii) a variety of participants’ profiles.

Based on the lists of villages covered by the community-led approach, villages were selected using stratified randomization based on geographical parameters. The latter are relevant in the case of Ebola (i.e., distance to the border of highly-affected countries). Stratification was done according to the location of each village from the border with Guinea and Liberia. For each district, a first sub-list of all villages between 0 and 30 km from a border, a second sub-list of all villages located between 30 and 50 km from a border, and a third sub-list of all villages located more than 50 km from a border (up to 80 km) were put together. After assigning each village name a specific number, village selection was done using the “RANDBETWEEN” function on Excel for each sub-list. The intention was to avoid any selection bias, i.e. the tendency to select villages where the community-led approach was considered successful.

**Fig. 2:**
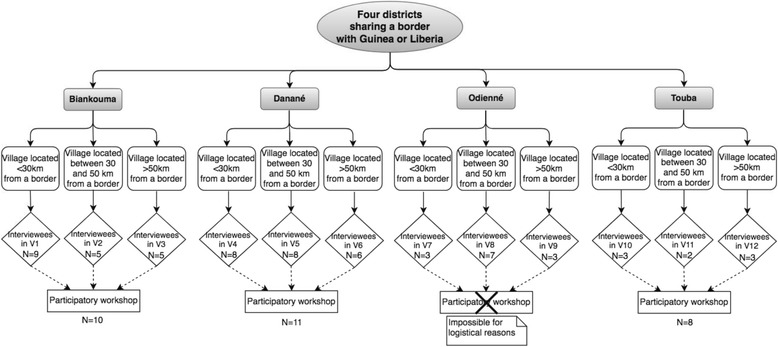
Data collection flowchart

As shown in Fig. 2, one village per sub-list was chosen, for each district.

Participants were included on a purposeful basis, according to their role: members of monitoring committees; community leaders who were not members of monitoring committees but who did participate in sensitization activities; ordinary adult community members; and CHWs’ supervisors.

### Data collection methods

Introduced by IRC’s community mobilization assistants, the research team went to health centers or “talking trees” to meet with the CHWs and village authorities. After presenting the study, participants were recruited based on suggestions made by monitoring committee members – independently of any influence by IRC.

Three pilot interviews were conducted in Biankouma district prior to regular data collection. The first data collection was carried out in Biankouma and Danané districts from September 9th to 19th, 2015. Two research assistants pursued data collection in the districts of Odienné and Touba from September 24th to October 3rd, and then transcribed all the data. In total, data was collected from 62 participants through 46 individual interviews and three focus group discussions. Details on the number of participants’ and their profile are provided in Table 2.

**Table 2:** Respondents’ profile in each of the 12 villages (V1 to V12)

	Biankouma			Danané			Odienné			Touba			Total
	V1	V2	V3	V4	V5	V6	V7	V8	V9	V10	V11	V12	
Ordinary Community Members (CM)	5	3	2	5	2	2		2					21
CHWs	1		1	1	1		1	2	1	2	2	2	14
Religious Leaders		1			1	1		1					4
Youth Representatives	1		1		1	2		1					6
Women Representatives	1					1			1				3
Village Authorities Representative		1		2	1			1		1			6
Chairs of health committes					1				1				2
Supervisors of health area	1		1		1		1						4
Traditional Healers							1					1	2
Total N participants per village	9	5	5	8	8	6	3	7	3	3	2	3	62
Total N participants per district	19			22			13			8			62

Besides interviews and focus group discussions, three participatory workshops were organized with CHWs. Community leaders and religious leaders were invited to these workshops. The workshops aimed at assessing their role as sensitizers and their views on the implementation of the sensitization activities. Each workshop involved: i) presenting the preliminary data collected in each district; ii) administering a self-assessment questionnaire with predefined sets of answers to rate sensitizers’ extent of adoption of alternative practices, their use of communication techniques, the difficulties they faced, and their recommendations; iii) representing results graphically on posters and discussing results; iv) translating posters’ content in local languages.

In total, among the 62 participants, 29 CHWs and community leaders (10 in Biankouma, 11 in Danané, and eight in Touba) were invited to a participatory workshop in their respected district capital city.

### Data analysis

Data was coded by the first author using the qualitative data processing software “TAMS Analyzer”. Based on standard coding techniques [8], the code book was revised by another person (the second author). The coded data was analyzed using thematic analysis [9].

Reliability of the results was ensured by researchers’ compliance with a transparent and systematic methodology, and the existence of a pre-test for interview guides in order to test participants’ understanding of issues. Data was collected by an independent research team (first author and two research assistants) in order to maintain constant neutrality during interviews, focus groups and participatory workshops. The team made sure that no undue influence from IRC was interfering with data collection. First, stratified randomization prevented any sort of influence in village selection. Second, IRC staff was absent from any direct interaction between participants and investigators.

The internal validity of the data collected was tested during participatory workshops. The participatory approach of this study enabled workshop participants to provide highly relevant contributions to this research. Their views and interpretation of the results is therefore considered in this report.

Results’ confirmability was tested during a pre-dissemination workshop held in the capital of Tonkpi region on September 22nd 2015. The preliminary results of data collected in Biankouma and Danané districts were presented to a wider audience (including regional and district-levels authorities, healthcare workers in charge of the selected health areas, and CHWs supervisors who were interviewed). Participants’ inputs during discussions were included in the analysis.

## Results

### Sources of information on Ebola

According to participants, government’s authorities played a leading role in delivering information about risks associated with the EVD. News about Ebola-related risks circulated through various other channels, evidenced by focus group discussions excerpt:

I2: We heard about it on TV, on the radio, and also through sensitizers.

(Focus Group Discussion, V1).^1^

The variety of information sources helped raise awareness about the disease:

Initially [the population] thought it was rumors. It is when they saw images from Guinea and when the government closed the borders that the people took the disease seriously. (Community Leader, V1).

### Program acceptance

Although it involved behavior change in daily practices requiring substantial efforts on the part of each community member, communities generally accepted the sensitization program. Main reasons of satisfaction primarily included: awareness of risks associated with Ebola, and protective effect of the program. The way sensitizers approached community members had a positive impact:

[The sensitizers] come to us with a fraternal and friendly approach to talk to us: this facilitates our understanding of the messages. (CM, V3).

All community member respondents showed strong appreciation of the sensitization efforts of their CHWs and leaders who worked to adapt EVD recommendations to their context. They recognized the risks posed by Ebola, which legitimized the sensitizers’ work:

[The] role of leaders and community health workers is to preserve the lives and health of populations. This is also why we offer no resistance (CM2, V3).

Furthermore, CHWs’ legitimacy was reportedly built on the fact that they represented the “link between administrative and health authorities and us, the communities” (CM2, V3).

**Fig. 3:**
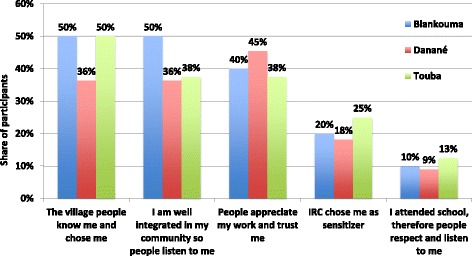
Most important reasons for sensitizers’ legitimacy across districts, according to participatory workshops participants. Percentages represent the share of participants rating the attribute in the top range, on a scale from 1 to 5, in terms of how important it is to them

The legitimacy and trustworthiness of sensitizers was linked to their personal history in the villages in which they operate – often having grown up in these villages. CHWs’ supervisors explained that CHWs’ legitimacy was based on the fact that members of the community had chosen them. Data collected during participatory workshops strengthened the relevance of choosing sensitizers well-integrated and respected in their communities in order to achieve program success. Among the predefined set of answers put forward to explain their legitimacy as sensitizers, answers like: *“The village people know me and chose me”* and *“I am well integrated in my community so people listen to me”* were most relevant by the majority of participants, as shown in Fig. 3. Many participants also considered “*People appreciate my work and trust me*” as highly relevant.

### Tools used for raising awareness

The most widely used sensitization strategies were door-to-door sensitization and mass sensitization. The former seems to have been preferred by the sensitizers, for fear of contamination with Ebola. Yet the main motivation for using the door-to-door technique was ensuring that households had clearly understood the prevention messages:

I ask questions, sometimes uncomfortable questions. And I take this opportunity to bring the right message, to explain in a better way. (CHW, V1).

Community members reported full satisfaction towards this sensitization strategy. During participatory workshops, sensitizers reported that the door-to-door strategy was the most effective: it gave the opportunity for each family member to ask questions without fearing what others might think. Small-group sensitization – in groups of women, young people, or within a church or mosque also reportedly worked very well. One technique was reported to be particularly effective: organizing drama or dance performances. Where finances permitted, these were organized in several areas (e.g., V3 and V9). In addition, handwashing demonstrations proved useful to make sure that households understand every step of this practice.

Sensitizers also used household inspection to detect unsafe practices. Their sensitization strategy was often based on initial observations, such as investigating houses’ courtyards. Sensitizers worked to find subtle ways to track unsafe behavior. This subtlety reflects commitment to the success of the community-led approach. The magnitude of more invasive strategies (e.g., spying) seemed to be confined to a very limited number of villages.

### Good understanding of unsafe practices

Recommended prevention practices were well understood. Community members that were interviewed reported applying them carefully. Each one of these respondents was able to cite at least three unsafe practices to avoid:

Eating bushmeat, shaking hands to greet, touching the body of deceased people, touching the sick. (Focus Group Discussion, V4).

Sensitizers corroborated these findings during interviews and participatory workshops. In the latter, when mentioning the difficulties encountered by sensitizers, the response “resistance of the people at the time of sensitization” was consistently ranked as the least relevant.

Community understanding of these sensitization messages probably facilitated compliance with alternative practices. In 11 of the 12 health areas, participants claimed that the majority of practices were effectively observed. CHWs’ supervisors also reported this information.

### Extent of adoption of alternative practices

The most readily adopted practice was handwashing with soap or bleach (or coal or ashes). Attendants of participatory workshops also reported that handwashing was the least difficult practice to implement in communities. Two sensitizers noted, however, that due to lack of material resources, like buckets and soaps, it was sometimes difficult for this to be practiced in all households. The sustainability of this practice could not be investigated at the time of data collection.

To prevent bushmeat consumption, it was recommended to cease hunting and replace bushmeat with fish or farmed meat. Initially, populations often offered some resistance. One reason for this could be that governments’ authorities had not issued any specific policy that would offer job alternatives to hunters, and/or subsidize other types of meat. Participants considered this a major challenge to implementing the recommendations, including at the pre-dissemination workshop. One participant, a high-ranking administration representative, acknowledged the lack of response from authorities and expressed hopes that authorities dealt with that issue more consistently.

Despite the challenges they could induce, the recommendations to replace bushmeat consumption were generally reported to be acceptable to people, as evidenced by this quote:

I, who speak to you, was a bushmeat seller. But because of Ebola, I do not sell bushmeat anymore. I asked my hunters to lay down their arms. (Community Leader, V1).

The main reason for the apparent acceptance of these recommendations was that it was possible to replace bushmeat with other foods that communities readily accepted, i.e. fish or beef. A few sensitizers conceded however that over time, efforts to comply with these recommendations against bushmeat consumption tended to decrease.

As it might be expected, contact with foreigners was more common in villages close to a border (within 30 km). Community members and sensitizers revealed that members of their own families often lived on the other side of the border, making it difficult to comply with the recommendation to avoid contact with them. The recommendation, however, was well respected in the villages located further away from the border.

When sensitizers, community members, and CHWs’ supervisors were asked which unsafe practice was the most difficult to give up, dead body management and greetings with hands were the most frequently mentioned. In many communities, raising one’s arms for greeting did not replace handshaking. This resistance could be explained by the traditions of greetings: traditions that were deeply rooted in these communities that attributed great importance to respect for others, especially towards someone known. Participants highlighted that the elderly were the most opposed to giving up handshaking. Still, nine respondents specifically mentioned that people had generally changed their behavior by not shaking hands when greeting. Yet it was conceded that over time, there was a tendency to “come back to usual forms of greeting” (Community Leader, V1).

Compared with greeting by raising arms, applying safety measures for the management of dead people’s bodies was of critical importance in order to mitigate EVD risks of infection. Despite sensitization efforts, these recommended practices also experienced enforcement difficulties in communities. Twenty-one participants in interviews and group discussions reported the challenges of changing their dead body management practices:

[…] because of the links that we have between us, it is difficult for us to abandon the bodies based on the idea that a health worker will take care of it. […] (Community member who has suffered a recent loss, V3).

When death occurred, despite the spread of recommendations (e.g., by the CHWs to the families of the deceased), these were often not applied due to a lack of supplies (e.g., gloves). Traditional and/or religious rituals organized in the case of death typically involve the presence of the elderly. Therefore some sensitizers were seen as lacking legitimate authority because of their relatively younger age. During workshops, participants noted that even if not all safety measures around dead body management were adopted, some of them were. They included: informing the health worker, wearing gloves when touching bodies, and abstaining from wearing the deceased’s clothing. Furthermore, according to sensitizers from one district, the number of days before burial of the deceased’s bodies was reduced from five to three or even two days. Even if this does not represent sufficient application, the reduction of time before burial demonstrated populations’ responsiveness and behavior change.

### Sensitizers’ perceptions of their activities

Sensitizers reported their satisfaction of playing the role of “knowledge brokers” on unsafe practices related to Ebola in the communities. First, sensitizers highlighted the contribution of the program to their skills’ enhancement in terms of EVD prevention and surveillance, which training and implementation of sensitization had developed. This program also had an empowerment effect:

I advise my family, my children. I feel that I’m useful. [...] I myself implement what I say, in order to represent a role model for my community (CHW, V1)

Four CHWs spoke about “being useful” to their community. The program strengthened their self-confidence and legitimacy.

During workshops, participants brought forward the idea that resistance to the implementation of practices – when it surfaced – would occur at the time of application, but almost never during sensitization activities. Communication links with community members, together with the respect towards sensitizers, forged links allowing messages to be delivered:

We use the local language so that parents understand better. (Community Leader, V3)

Applying alternative practices was more problematic, in particular those related to dead body management and greetings. Besides challenging community traditions, another reason for this non-compliance was given: lack of material and financial resources available to populations.

### Recommendations by sensitizers and workshops’ participants

**Fig. 4:**
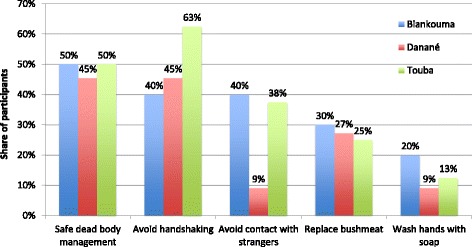
Most difficult unsafe practices across districts to adopt, according to participatory workshops participants. Percentages represent the share of participants rating the attribute in the top range, on a scale from 1 to 5, in terms of how important it is to them

Participatory workshops provided occasions to compare results about perceived compliance with alternative practices across districts. Participants ranked the importance of a list of items (i.e., items of response directly stemming from preliminary analysis of qualitative interviews). As exhibited in Fig. 4, in Touba, handshaking was reportedly more difficult to give up than in other districts. Figure 4 also shows that in Danané sensitizers reported that avoiding contact with a stranger was not difficult to comply with, as compared to the other two districts. This may be due to the fact that in Danané, most sensitizers attending the participatory workshop came from villages located further away from the border. Apart from handshaking and contact with a stranger, results were roughly similar across the three districts.

In order to increase people’s adoption of alternative practices, sensitizers primarily offered to strengthen sensitization activities. As for dealing with reluctant participants, 15 sensitizers emphasized strengthening sensitization, while three were willing to make use of threat. In the case of hunting or consumption of wild animals, two CHWs mentioned urging those who opposed greater resistance to change their behavior, by pointing to the risks of non-compliance. At times, warnings were repeated – but they never replaced the roles played by sensitization messages. As a result, CHWs rarely reported cases of conflict. A CHW often played both “health official in charge of surveillance” and “community sensitizer”: this contributed to CHWs refraining from following through their threats. They avoided any risk of social confrontation.

Regarding the limited adoption of safe body management practices, a CHW made a particularly interesting comment:

I ask them to try to implement the recommendations and to call me in case of death. In turn, I’ll go see the [qualified health worker]. Sometimes subtly, I ask them if the deceased person had symptoms like those of Ebola. (CHW2, V12)

This sensitizer sought to understand the situation in a subtle manner, i.e. taking into account cultural sensitivities and the context of grief, and weighing whether the deceased was likely to represent a suspected case, and/or the reasons behind non-compliance with the recommendations.

CHWs’ supervisors (i.e., qualified health workers) reported that CHWs regularly transmitted (by SMS) instances of non-compliance and suspected cases of deceased persons. Qualified health workers then had to decide how to respond. The CHWs served as intermediary actors, enabling them to be protected from being held accountable, thereby preventing any risk of retaliation.

In general, according to sensitizers, sensitization efforts should be sustained in communities in order to capitalize on achievements. A number of recommendations were suggested: providing mobility equipment to reach out to community members (e.g., providing a bike and a pair of boots in each village), providing more material resources to support the change in practice (e.g., providing a few pairs of gloves and masks to secure compliance to alternative practices in case of death), and strengthening training for ensuring better sensitization.

## Discussion

### Contribution of the community-led prevention program

The investigation highlights several positive lessons learned from the implementation of this program. First, the program increased and scaled-up routine handwashing practices in the four districts. According to study participants, sensitization also reduced bushmeat consumption and wild animal hunting, though not completely putting an end to this practice. Finally, the messages transmitted to the communities reportedly helped to reduce the incidence of other unsafe practices, including contact with foreigners (even though imperfectly in border areas), greeting by shaking hands, and unsafe dead body management. These results need to be contrasted with those of other countries with community sensitization activities. Thus far, there have been positive reports from Liberia [10], but more disappointing reports from Guinea [11, 12] and Sierra Leone [13]. Other studies have reported that poor outcomes from sensitization might be due to mistrust towards health workers and the health system in general [14–16].

Furthermore, in Western Côte d’Ivoire, sensitization efforts have clearly helped to complement messages delivered by government’s authorities and the media (radio, TV). According to participants, this combination largely contributed to understanding the risks associated with different practices. Interestingly, it was reported to us that in cities (like in Danané, where people had not received similar sensitization), rumors around Ebola (i.e., conspiracy theories, negation of disease existence, etc.) circulated far more than in rural areas. During village investigations, this issue came up only once: a community member had listened to a radio program from Liberia disseminating false messages on Ebola.

The clarity and appropriateness of messages provided during sensitization activities in villages likely had the effect of reducing rumors and misinformation around the virus. This finding would represent an important achievement given the profile of these geographical areas (Tonkpi, Kabadougou and Bafing regions), faced with an environment prone to recurring anxiety and violence (due to frequent sociopolitical troubles), but also given the low socioeconomic status of the people of these regions [17, 18]. Therefore, in spite of a complex social atmosphere, this finding would indicate that it is possible to decrease misinformation: ownership of accurate Ebola-related messages issued by CHWs and community and religious leaders may have reduced mistrust and misinformation. This is consistent with Cohn & Kutalek’s accounts about the value of community engagement in such context [19]. Yet, outside of Côte d’Ivoire, in other geographical areas more directly affected by the EVD and showing similar socioeconomic and political profiles (such as, on the other side of the borders with Liberia and Guinea), misinformation persisted [12, 15, 16].

Community leaders might be better positioned to develop and diffuse “health communication strategies […] tailored to the local circumstances” [20]. This study shows that relying on community leaders to spread sensitization messages may have been the key to program acceptance. Because sensitizers were well integrated and respected in their communities, they were perceived as legitimate and therefore were able to promote healthier behaviors. This choice was conducive of effective behavior change. This is congruent with recent findings from a neighboring country (Ghana) on the role of community and traditional leaders, who “serve as the pivot around which social and human capital of the communities revolve in the developmental process” [21]. It is also in line with broader literature on the pivotal role of CHWs during the Ebola epidemic [22].

The strategy was also beneficial for the sensitizers themselves. It helped strengthen their own sense of legitimacy and their ability to promote behavior change in their communities beyond this Ebola-prevention program. Evidence of CHWs and community leaders’ engagement for the successful implementation of the program was perceived by the research team as being extremely strong, for two main reasons.

First, their involvement in this program has strengthened their consciousness of legitimacy in the villages (e.g., references to the feeling of “being useful to the community”), and thereby their intrinsic motivation, which should be sustained in the long-term. Second, as evidenced by the extracts reported in the Results section, all categories of sensitizers demonstrated leadership needed to ensure that messages were respected. For example, Catholic priests officiating in this area also took the initiative to provide churches with hygiene devices (a bucket with soap) at the entrance of churches. Besides the classic sensitization techniques for which they received training by IRC, they deployed multiple and subtle strategies (e.g., adapting the messages to their own observations of households insufficiently changing their behaviors). In particular, community health workers successfully managed to adjust and articulate their dual role of sensitizer and monitoring surveillance in communities. They understood that monitoring the implementation of recommendations did not involve spying or denouncing but instead called for subtle strategies, particularly when dealing with mourners. In Guinea, this feature did not appear to take place: authors reported distrust “between communities and those seeking to control the epidemic”, arguing that it “largely contributed to the reluctance of the communities to participate and contribute to the effort” [16]. One could therefore interpret the absence of CHWs reporting to the authorities as representing the sine qua non condition of maintaining trust in the community and beyond.

Second, additional evidence of ownership by the sensitizers was identified: this could also be considered an equally unexpected legacy of the program: once they were trained by IRC staff, monitoring committee members in turn trained community leaders as “support sensitizers”, disseminated in different neighborhoods. The use of support sensitizers ensured that messages reached the entire population, and each household. Then, in turn, sensitized individuals themselves became sensitizers through what can be called a snowball effect: some reported having delivered messages to their relatives and friends. This self-created “sensitization chain” was particularly beneficial to the effective behavior change that was witnessed afterwards. Indeed when everyone becomes a sensitizer, the effect of peer influence plays an instrumental role in changing practices, mitigating the “condemnatory” and short-termist effects produced by simple administrative bans [23].

### Implementation challenges and imperfections of IRC’s community-led approach

This program promoting behavior change was implemented after the national government had announced bans to avoid any risk of EVD infection. According to MSF/Epicentre, economic and social consequences of these decisions were numerous [24]. This study confirmed that these administrative bans created many problems: respondents pointed to higher food prices due to the closing of markets in border villages. These bans also resulted in the “dislocation” of some families as some lived on opposite sides of Côte d’Ivoire’s borders with Liberia and Guinea. These bans also brought challenges to the implementation of the community-based approach. The main issue was that the first messages about EVD that communities heard were these prohibitions: people were told not to eat and hunt wild animals, not to greet by shaking hands, etc. For these reasons, despite the community-led sensitization efforts, alternative practices were infrequently mentioned by community members, apart from those of handwashing with soap, and fish consumption to replace bushmeat. For example, it was only in the district Biankouma that raising one’s arm to greet was mentioned. It is likely that sensitizers themselves chose replacement strategies that they had identified as most appropriate given the context. Perhaps they did not necessarily see the need to hear messages from “outside” (i.e., from IRC) informing them of the different alternative practices. In fact, this was the very nature of the “community-led” strategy: ultimately sensitizers and community members were best placed to identify themselves their own ways to adapt to the situation.

The program could have better considered practical aspects, in order to facilitate the dissemination of messages. Sensitizers’ needs in terms of mobility and equipment came out very clearly in discussions with study participants. Some unsafe practices persisted: the common explanation was that they were challenging local traditions and values. Yet sensitizers reported that with additional material resources (e.g., gloves, bicycles etc.), the turnout might have been different. Given that dead body management was noted to have driven Ebola spread in nearby countries, more emphasis on the conditions of implementation of safe, culturally and socially acceptable practices, would have enabled better community ownership. Adjusting sensitization messages, making them more targeted and tailored, should further reduce the extent of these unsafe practices.

### Opportunity for capitalization

The outcomes of this program appeared to be sustainable. Attention could be paid to sustaining and scaling up handwashing, which has benefits beyond Ebola. Improved handwashing could reduce other diseases incidence (diarrhea, cholera, respiratory pathogens etc.). Recognition of this potential was clear in discussions with the CHWs and their supervisors.

In addition, the feelings of legitimacy and ownership of the program should continue to be reinforced. Given the success of sensitization efforts, it would be useful to capitalize on this strong commitment. For example, apart from Ebola, it would be possible to assign the sensitizers (and not just CHWs) new missions to further promote healthy behavior change. However, these new attributions should be accompanied by proper incentives – including material incentives. Intrinsic motivation, which currently stimulates them – and was strengthened by this program (i.e. they clearly felt recognized and useful) – should, however, constitute the foundation of new programs. Future research could further explore these aspects.

### Limitations

The participatory approach was partially implemented: co-creation of knowledge [25] was done with only a selection of stakeholders’ categories. For instance, ordinary community members were not invited to participatory workshops that allowed for dissemination and specification of some preliminary results, and also generated some additional data. Given the focus of the analysis on implementation, it was decided to involve sensitizers in order to focus on the experience and potential improvements of sensitization.

For logistical reasons, it was not possible to organize a participatory workshop in Odienné – a region hard to reach by car. For financial reasons, it was also impossible to invite all of the ordinary community members that were interviewed to the pre-dissemination workshop that was held in Man. The CHWs’ supervisors that were interviewed did participate in the pre-dissemination workshop. In the end, this participatory research primarily involved CHWs, community and religious leaders.

Lastly, this study being qualitative, there were no control communities (i.e., where no sensitization took place), and there were no pre-post measurement of behavior involved in the study. Therefore, attribution of the reported changes to the sensitization program can only be interpreted in the context of participants’ perceptions.

## Conclusion

This study demonstrates that sensitization efforts by well-integrated and well-respected community and religious leaders, can lead to behavior changes that are essential to people’s health, even in a complex socioeconomic and political context – and even in preparation for a risk not yet present. Even though Ebola rapidly spread through Guinea, Liberia, and Mali, the epidemic never reached Côte d’Ivoire. The strong involvement of community sensitizers and their demonstrated leadership in Western districts of the country could be a part of this major success.

Lessons learned from the community-led strategy could be applied to future disease outbreaks, a risk given the previous discovery of zoonotic Ebola transmission in Côte d’Ivoire and the persistence of other diseases of importance like yellow fever. The lessons could also be applied more widely than epidemic prevention. Health system strengthening programs should involve more meaningfully CHWs, community and religious leaders, and find sustainable solutions to strengthen their motivation.

## Abbreviations

CHW: Community Health Worker
CM: Community Member
EVD: Ebola Virus Disease
IRC: International Rescue Committee
USAID: United States Agency for International Development

## Footnotes

1 All translations by the author of this paper unless otherwise noted.
